# Effectiveness of Perineal Protection Devices in Reducing Birth-Related Perineal Trauma: A Systematic Review and Meta-Analysis of Randomized Controlled Trials and GRADE Assessment

**DOI:** 10.1007/s00192-025-06364-y

**Published:** 2025-11-19

**Authors:** Salma Allam, Omar Khalid Samir Abdelkader, Rana Mansour, Nour Elsayed, Hind Elshiekh, Joudi Tarabishi, Sara Mohammad Hegazy, Amr Arafa, Ahmed Kertam

**Affiliations:** 1 Faculty of Medicine, Galala University, Galala City, Suez Egypt; 2 Port Said University Faculty of Medicine, Port Said, Egypt; 3 Faculty of Medicine, Zagazig University, Zagazig, Egypt; 4 Department of Obstetrics and Gynecology, University of Aleppo, Faculty of Medicine, Aleppo, Syria; 5 Faculty of Medicine, New Mansoura University, New Mansoura, Egypt; 6 Faculty of Medicine, Alexandria University, Alexandria, Egypt; 7 Tu Lab for Diagnostic Research, Yale School of Medicine, New Haven, CT USA

**Keywords:** Perineal trauma, Vaginal delivery, Perineal protection device, Randomized controlled trials, Birth injury, Obstetric anal sphincter injury

## Abstract

**Background:**

Perineal trauma during childbirth is a common complication with significant short- and long-term maternal consequences. Perineal protection devices (PPDs) have been developed as a potential preventive strategy, yet their clinical effectiveness remains under evaluation.

**Aim:**

To assess the effectiveness of perineal protection devices in reducing the incidence and severity of birth-related perineal trauma.

**Methods:**

A systematic review and meta-analysis of randomized controlled trials (RCTs) was conducted. Eligible studies were identified from PubMed, Scopus, Web of Science, and Embase. Primary outcomes included rates of intact perineum, perineal tears (grade ≥ 2), labial tears, episiotomy, and Apgar scores. Secondary outcomes included adverse effects and feasibility. The Cochrane Risk of Bias tool 2 and GRADE assessment were used to evaluate study quality.

**Results:**

Five RCTs involving 2331 participants were included. PPD use was associated with a significantly increased rate of intact perineum (RR 1.41; 95% CI 1.18–1.69; *p* < 0.001). No significant differences were found for grade 1 (RR 1.05; *p* = 0.48), grade 2 (RR 0.92; *p* = 0.32), or grade 3–4 perineal tears (RR 0.76; *p* = 0.26). Labial tears showed no significant reduction overall (RR 0.90; *p* = 0.64), but sensitivity analysis revealed a benefit after excluding one study (RR 0.72; *p* = 0.02). No significant differences were found in episiotomy rates (RR 0.96; *p* = 0.62) or Apgar scores < 7 at 5 min (RR 0.99; *p* = 0.93).

**Conclusion:**

PPDs appear safe and effective in increasing intact perineum rates without adverse neonatal outcomes. Further large-scale, standardized trials are needed to confirm their role in obstetric practice.

**Graphical Abstract:**

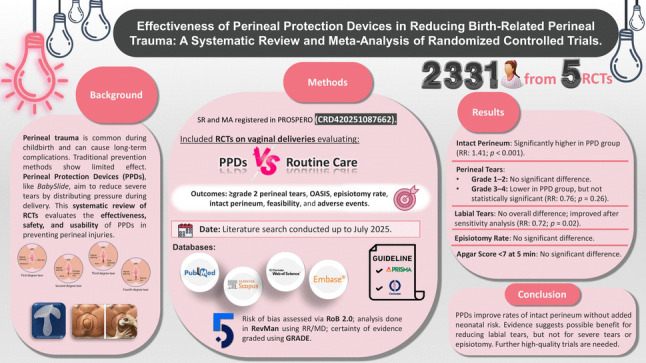

**Supplementary Information:**

The online version contains supplementary material available at 10.1007/s00192-025-06364-y.

## Introduction

Perineal trauma, defined as injury to the perineum or female genital tract during childbirth, may occur spontaneously or as a result of medical intervention [1, 2]. These injuries are classified by severity: first-degree tears involve only the skin and mucosa; second-degree tears extend to the perineal muscles; third-degree tears involve the anal sphincter and are subdivided into 3 A (less than 50% of the external anal sphincter [EAS] torn), 3B (more than 50% of EAS torn), and 3 C (involving both the EAS and the internal anal sphincter [IAS]); while fourth-degree tears extend through both sphincters and the anorectal epithelium [3]. Perineal injuries are highly prevalent, particularly among primiparous women [4]. A recent study reported episiotomy in 68.3% of vaginal deliveries, with spontaneous lacerations in 16%, resulting in an overall perineal trauma rate of 84.3%. The incidence of perineal lacerations was 27.5% in primiparas versus 3.5% in multiparas, with overall trauma affecting 98.5% and 71.4% of these groups, respectively [5]. Such injuries are associated with a range of short- and long-term complications, including urinary and fecal incontinence, dyspareunia, and delayed resumption of sexual activity [6]. While obstetric anal sphincter injuries (OASIS) are the most severe form of perineal trauma and a leading cause of anal incontinence and long-term morbidity [7, 8], less severe injuries such as second-degree tears are more common and contribute substantially to maternal morbidity. These include impaired sexual function [9, 10], dyspareunia [11], increased risks of urinary incontinence [12] and pelvic organ prolapse [13], and heightened postpartum pain [14]. Alarmingly, data from the Swedish National Quality Register of Gynecological Surgery show that 15% of women with second-degree tears reported anal incontinence 1 year after delivery [15]. In 2019, the incidence of OASIS in Sweden was 2.6%, rising to 4.6% among primiparous women [16], while second-degree tear rates remain inconsistently reported, ranging from 35 to 78% [17, 18]. Preventive strategies such as perineal massage, warm or cold compresses, and manual perineal support have been explored [4], but a 2017 Cochrane review found no significant effect on second-degree tear reduction [19]. Some interventional programs using specialized manual maneuvers have shown reduced sphincter injury rates [20], yet the American College of Obstetricians and Gynecologists has concluded that current evidence is insufficient to recommend any manual technique [21]. In response to this gap, various perineal protection devices (PPDs) have been developed to reduce pressure on the perineum during crowning. These devices are designed to distribute pressure over a broader surface area and protect the vaginal mucosa [4]. One such example is the BabySlide (Karo Healthcare, Sweden), evaluated in a randomized controlled trial by Lavesson et al. [22]. Although no significant reduction in OASIS was found, the study reported increased rates of intact perineum and fewer severe perineal tears. The BabySlide can also facilitate in situ episiotomy and guide proper incision angles. Since then, the device has undergone minor modifications in size to improve fit and usability.

Given the clinical relevance of perineal trauma and the emerging role of PPDs in obstetric care, this study aims to systematically review randomized controlled trials to critically evaluate the effectiveness, safety, and feasibility of PPDs in reducing birth-related perineal injuries. By synthesizing existing RCTs, this review seeks to strengthen the current evidence and guide future practice and research on the use of such devices.

## Methods

### Protocol Registration

We registered the protocol for this systematic review and meta-analysis with the International Prospective Register of Systematic Reviews (PROSPERO) under the registration number (CRD420251087662).

### Eligibility Criteria

We included studies in our review if they met all of the following criteria: studies involving women undergoing vaginal delivery (spontaneous or vacuum-assisted), including both primiparous and multiparous women; studies that evaluated the use of a perineal protection device during the second stage of labor to reduce birth-related trauma; studies comparing women who received routine care without the device, which may include standard manual perineal support techniques or hands-on/hands-off approaches; and studies reporting at least one of the following outcomes: grade ≥ 2 perineal tears, labial tearing, vaginal tearing, anal sphincter injury (grade 3–4), episiotomy rate, intact perineum (no tears), feasibility, or adverse effects of device use. Non-English manuscripts were translated using professional translation tools or native-language reviewers when necessary. We excluded studies if they met any of the following criteria: observational studies, case reports, reviews, editorials, or conference abstracts without full data, or non-randomized trials; studies on cesarean section deliveries; studies assessing manual techniques only without any device; studies using non-device-based perineal supports (e.g., massage, compresses only); or animal studies or studies conducted on non-human subjects.

### Literature Search and Keywords

For this meta-analysis, we conducted a systematic search of the literature on the four electronic databases, PubMed, Scopus, Web of Science, and Embase from their inception until 1 July 2025. Additionally, we screened the reference lists of the included studies to capture any additional relevant articles. We used the following search terms to find relevant studies: (“Perineum” OR perineal OR perineum) AND (“Wounds and Injuries” OR trauma OR tear* OR laceration* OR injury OR “obstetric injury” OR “perineal trauma” OR episiotomy OR “anal sphincter injury”) AND (“Protective Devices” OR device* OR “perineal support device*” OR “perineal protection device*” OR “birth support device*” OR “perineal guard” OR “Epi-No” OR “vaginal balloon” OR “silicone ring” OR “perineal distension device”) AND (“Labor, Obstetric” OR “Childbirth” OR delivery OR birth OR “vaginal delivery”). We applied this search strategy to all four databases and included the full search details and results in Supplementary Table 1.

We followed the PRISMA guidelines. We used the Cochrane Handbook as a guide to conduct and report this systematic review and meta-analysis [23, 24].

### Data Collection Process and Items

**Fig. 1:**
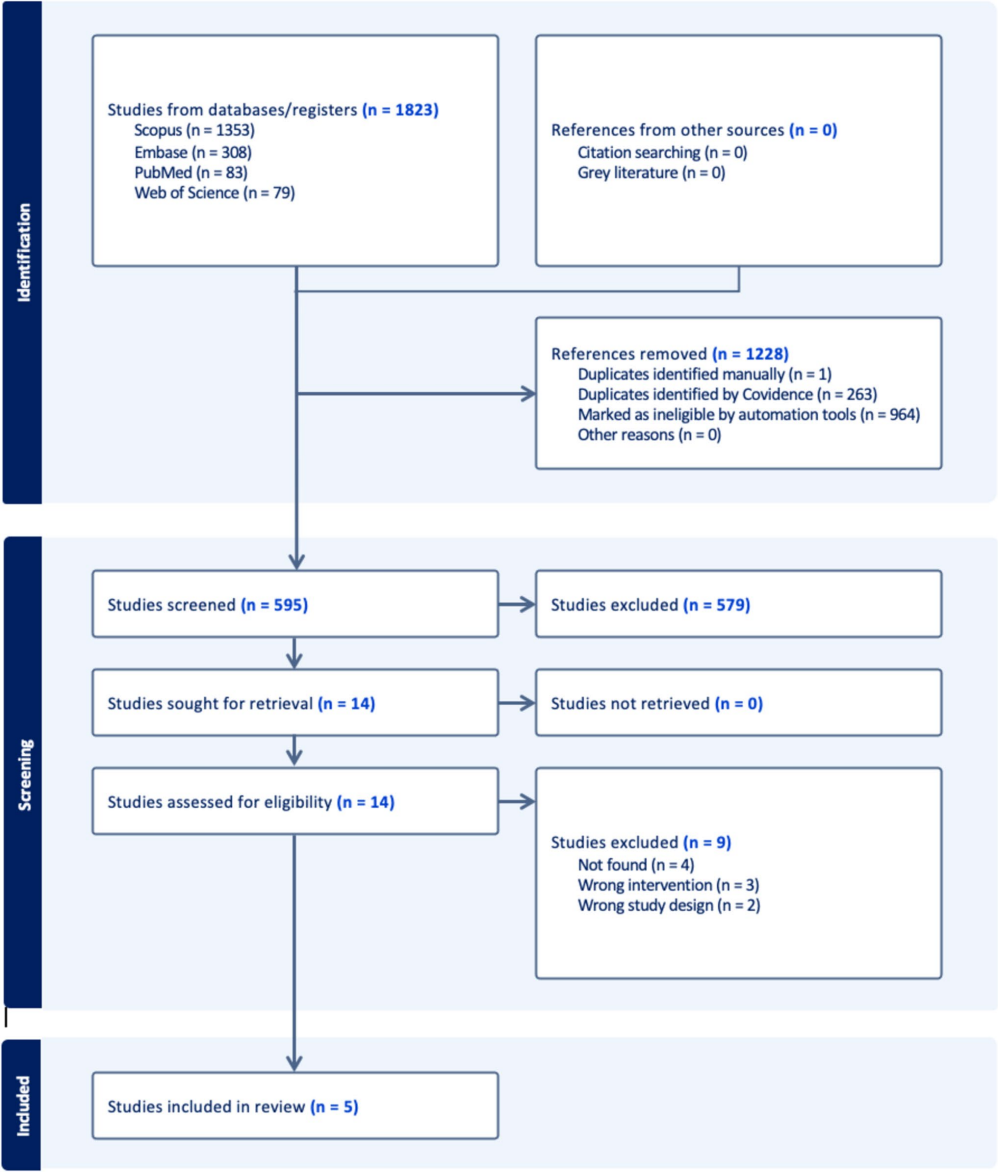
PRISMA flowchart for the study selection process and databases search

We uploaded all studies into Covidence and used its tool to remove duplicates. Then, we screened the studies in two steps. The first step was that three reviewers independently screened the titles/abstracts of all studies. The second step was to screen the full texts of potentially relevant studies based on the eligibility criteria. A fourth reviewer resolved any disagreements. The screening process is presented in the PRISMA flowchart in Fig. 1.

### Data Collection Process and Data Items

‏Two reviewers independently extracted the data from each study using a standardized data extraction sheet. The extracted information included: (1) summary of the included studies (first author, publication year, title registration number, study design, country, sample size, intervention, primary outcome, secondary outcome, inclusion criteria, exclusion criteria, and main findings); (2) study populations characteristics (study ID, number of patient in each group, age, gestational age, second stage of labor, any instrumentation, epidural use, episiotomy use, vacuum extraction); (3) risk of bias domains; and (4) outcome measures (the primary outcomes were the perineal tear grades 1–4, vaginal tears, labial tears and the secondary outcomes included obstetric anal sphincter injuries (OASIS), episiotomy rate, intact perineum, feasibility/adverse events and Apgar scores). Any disagreements were resolved by a third reviewer.

### Risk of Bias Assessment

Two reviewers independently assessed the quality of each included study using the Cochrane Risk of Bias 2.0 (RoB2) tool across its seven domains, as recommended in the Cochrane Handbook for Systematic Reviews of Interventions. Any disagreements were resolved by a third reviewer.

### Statistical Analysis

We used Review Manager (RevMan) version 5.4. software to perform all statistical analyses. For dichotomous outcomes, we calculated risk ratios (RR), while for continuous outcomes, we computed mean differences (MD), both with 95% confidence intervals. A *p* value < 0.05 was considered statistically significant. To assess heterogeneity among studies, we used both the I^2^ statistic and the chi-square test. If heterogeneity was substantial (I^2^ > 50%), a random-effects model was applied; if not, a fixed-effects model was used. We included only five studies; therefore, we did not assess publication bias using funnel plots or statistical tests, and instead, we limited our assessment to conducting a ROB assessment and assessed the certainty of evidence for each outcome using the GRADE (Grading of Recommendations Assessment, Development and Evaluation) approach across key domains: risk of bias, inconsistency, indirectness, imprecision, and publication bias as shown in Supplementary Table 2.

## Results

### Study Selection

We retrieved 1823 records from four databases during our literature search process; 1353 from Scopus, 308 from Embase, 83 from PubMed, and 79 from Web of Science databases. A total of 264 records were removed as duplicates; 263 by covidence, and one manually. Following the removal of duplicate and ineligible records, 579 records were screened. After the title and abstract screening, 14 full-text articles were considered eligible. Of them, five studies were included in the systematic review and meta-analysis. The reference lists of these studies were manually searched, but no additional relevant articles were identified. The study selection process is presented in the PRISMA flow diagram in Fig. 1.

### Study Characteristics and Quality Assessment

**Fig. 2:**
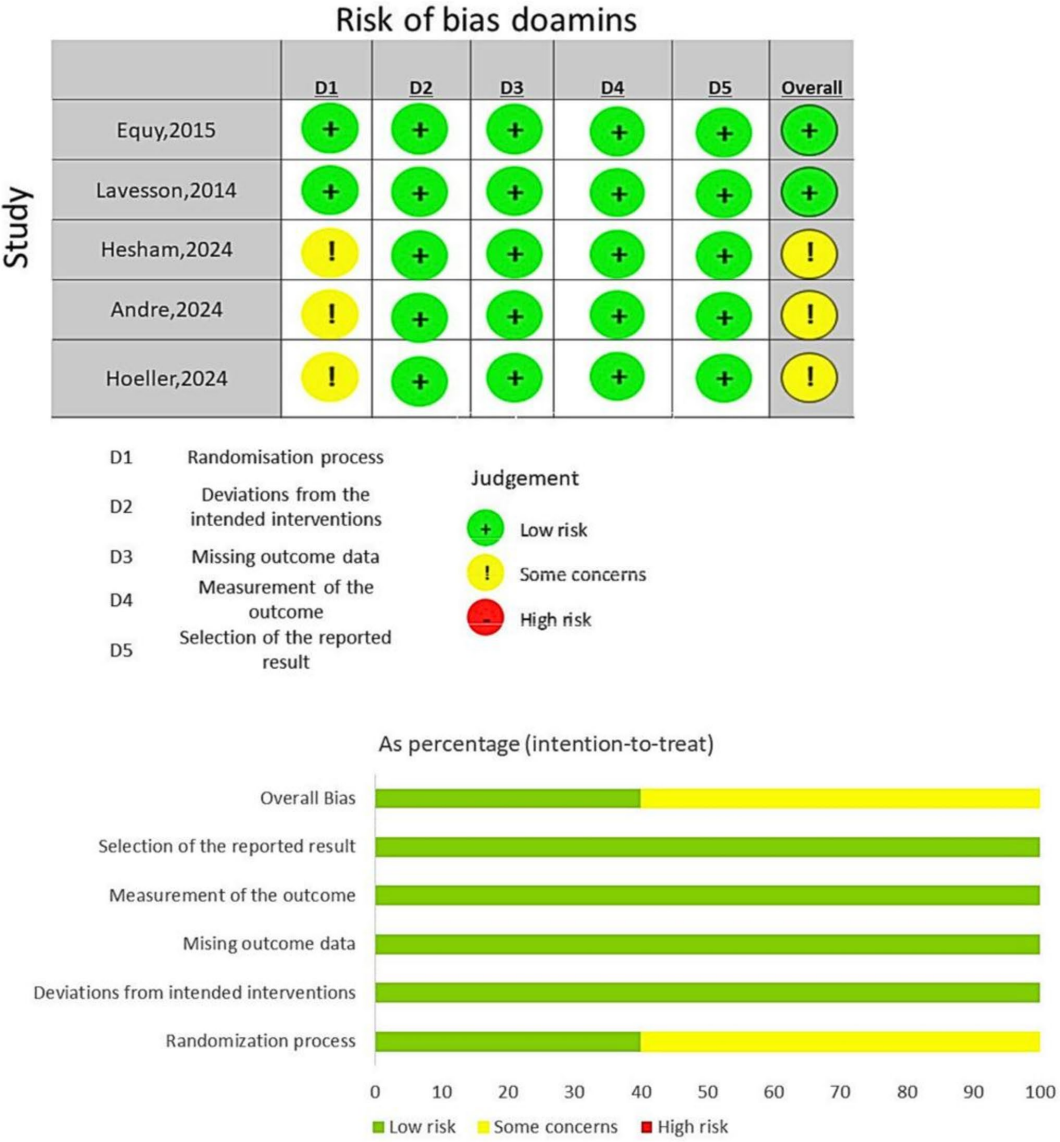
ROB 2.0 graph and summary for the included studies

Five studies were included in the systematic review and meta-analysis, all of which were randomized controlled trials (RCTs), with a total sample size of 2331 patients. In all studies, patients were assigned to either an intervention group receiving perineal protection or a control group receiving standard care or routine clinical practices during vaginal delivery. A summary characteristic of the included studies is presented in Table 1, and the baseline characteristics of the study populations are presented in Table 2 [4, 22, 25–27]. The risk of bias assessment of the included studies is presented in Fig. 2, according to the Cochrane risk of bias tool 2 (ROB2). Among the five RCTs, two studies were assessed to have low risk of bias, and the remaining three studies have some concerns regarding risk of bias. We used the GRADE system to assess the level of evidence for each outcome. The intact perineum and perineal tear grade 1 outcomes were both rated as moderate evidence certainty. For intact perineum, evidence from three RCTs (1768 participants) showed a significant increase with the intervention compared to the control group (RR 1.41, 95% CI 1.18–1.69; *p* < 0.001). However, certainty was downgraded to moderate due to serious inconsistency, as indicated by a substantial I^2^ of 60%. For perineal tear grade 1, the effect estimate was RR 1.05 (95% CI 0.92–1.21) with no heterogeneity (I^2^ = 0%) across three RCTs (879 participants). The certainty was downgraded to moderate due to serious imprecision, as the wide confidence interval for the effect estimate crossed the null value.

**Table 1:** Summary of the included studies

Last name, year	Trial registration number	Study design	Country	Sample size	Intervention	Primary outcome	Secondry outcomes	Inclusion criteria	Exclusion criteria	Main finding
Equy, 2015	NCT01058200	multicenter prospective randomized controlled open clinical trial	France	668	vacuum extraction using the iCup or usual Drapier-Faure metallic cup	composite including both the risk of cup dysfunction responsible for a clinical impact (prolongation of delivery and eventually change in mode of delivery) and the most frequent maternal and neonatal harms The precise criteria were as follows: the use of other instruments after attempted vacuum extraction, caesarean section after attempted vacuum extraction, three detachments of the cup (considered as failure in French recommendations [4]), episiotomy and perineal tears; and for the new-born: caput succedaneum (swelling of the scalp), cephalohaematoma (an effusion of blood beneath the periosteum of the skull). If one or more of these elements were observed an “event” was considered to have occurred; otherwise “no event” was recorded Obstetric data were collected by the obstetrician but were objective well-defined criteria. Pediatric data were recorded by an independent pediatrician A composite criterion was chosen because we wanted to include both neonatal and maternal elements in judging the success of the intervention. Nevertheless, we needed to find a balanced compromise between similar severity and the frequency of each element The components of this composite endpoint represent the vast majority of clinical risks of instrumental vaginal delivery using a ventouse [3]. Furthermore, the advantage of using a composite endpoint was to be able to immediately assess the potential superiority of the instrument with a sufficient number of events without requiring an excessively large number of patients for the trial	maternal and neonatal outcomes that are rare, are minor, or are independent of the type of device used:neonatal lesions: minor scalp injuries, Apgar score < 7, pH < 7.20, anaemia (haemoglobin < 14.5 g/dl), jaundice (bilirubin > 150 μmol/l), transfer to NICU	Women were included if they were aged between 18 was ≥ 36 + 0 weeks at delivery and if instrumental extraction was indicated	Refusal to participate Under legal protection or deprived of liberty by judicial or administrative decision Final absence of indication for vacuum extraction (i.e., spontaneous delivery occurred or decision changed) Operator’s decision against vacuum extraction due to newly appearing contraindications	While the disposable cup had more detachments and extraction failures than the standard metallic cup, this innovative disposable device had the advantage of fewer perineal injuries
Lavesson, 2014	NCT01533467	multicenter open randomized controlled trial (RCT)	Sweden	1148	perineal protection device used vs perineal protection device was not used	incidence of vaginal and perineal tears (1st to 4th degree tears) and adverse effects on the parturient and newborn	-	delivery with cephalic presentation,age of more than 18 years and an understanding of both oral and written information in Swedish	Per prot ocol analysis: Excluded, device was not us ed n = 74◆ 1 st and 2o nd degree te ars = 65◆ ASR = 9, Women undergoing emergency caesarean section,Medical journals were inadequately completed,	The perineal protective device significantly reduced the incidence of first- and seconddegree tears in the vagina and perineum during vaginal birth and also significantly increased the number of parturients with a fully intact posterior commissure. No significant reduction of ASR and no negative effects of the device were observed
Hesham, 2024	-	multicenter, randomized controlled Pilot trial	USA	214	intrapartum electromechanical pelvic floor dilator (IPD) vs standard-of-care labor management	The primary effectiveness endpoint was the rate of pelvic muscle injury, defined as complete LAM avulsion diagnosed by transperineal ultrasound of the pelvic floor anatomy at 3 months postpartum	rates of partial LAM avulsion, perineal lacerations, obstetric anal sphincter injuries (OASIS) [20], conversion to C-section owing to arrest of the second stage of labor, and duration of the second stage of labor. The primary safety outcome was incidence of adverse events (AEs) through 3 months postpartum	nulliparous individuals aged ≥ 18 years planning first singleton vaginal delivery at term (> 36 weeks), with willingness to receive epidural anesthesia prior to randomization	fetal chromosomal or structural anomalies, local or systemic infection, maternal history of connective tissue disorders or neurological disease that could impact delivery, or unresolved intrapartum category 2‒3 fetal heart tracing prior to randomization	The pelvic floor dilator device significantly reduced the incidence of complete LAM avulsion in nulliparous individuals undergoing first vaginal childbirth. The dilator demonstrated an acceptable safety profile and was well received by recipients. Use of the intrapartum electromechanical pelvic floor dilator in laboring nulliparous individuals may reduce the rate of LAM avulsion, an injury associated with serious sequelae including pelvic organ prolapse
Andre, 2024	-	single-center RCT	Sweden	92	perineal protection device used vs routine manual perineal support	grade of perineal tear	vaginal and labial tearing	gestationalage > 37 0/7 weeks, primipara, cephalic presentation, age ≥ 18 years, and understanding of written and spoken Swedish	planned oracute emergency cesarean delivery	The use of a perineal protection device reduced the risk of grade ≥ 2 perineal tearing by 60%and labial tearing
Hoeller, 2024	-	prospective,randomized controlled interventional trial	Switzerland	209	Perineal protection device ((BabySlide, Karo Pharma AB, Stockholm, Sweden)	rate oflow-grade perinealand/or vaginal tears in the posterior compartment	Secondary outcome swere the rate of high-grade perineal tears and episiotomies,the feasibility of the device,and its safety for the mother and child	patients were eventually included in fulfilled the screening criteria, none of the exclusion criteria were met, and the gestational age was ≥ 36 + 0 weeks	maternal connective tissue disease, vaginal, prior to randomization than birth tear repair, previous pelvic floor or incontinence surgery, female genital mutilation, intrauterine fetal demise, external fetal malformations with additional space requirements within the birth canal and therefore potential impact on the course of birth, use of the Epi-No perineal trainer before birth, and allergies against any material of the device	The perineal protection device was not able to reduce the primary composite outcome of the study among women who gave birth by vacuum assistance. Although this was not the primary end point, a lower rate of mediolateral episiotomies was observed when the device was used correctly. As a limitation, difficulties with the application of the device occurred in 33% of cases. Nevertheless, no adverse events in association with the device were observed for either mother or child in our cohort

**Table 2:** Baseline characteristics of the included studies

Last name, year	Number of patients in each group		Age (mean years ± SD)		Gestational age (wk)		Second stage of labor (min)		Any instrumentation use, % (*n*/*N*)		Epidural use, % (*n*/*N*)		Episiotomy, *n* (%)		Vaccum extraction, *n* (%)	
	Intervention	Control	Intervention	Control	Intervention	Control	Intervention	Control	Intervention	Control	Intervention	Control	Intervention	Control	Intervention	Control
Equy,2015	295	283	28 ± 5	29 ± 5	40 ± 1	40 ± 1	NA	NA	295	283	294 (99.7%)	278 (98.2%)	NA	NA	295	283
Lavesson,2014	546	552	30.65 (4.426)	31.3 (4.75)	38.55 (1.475)	38.8 (1.31)	121.25 (58.197)	123.25 (58.134)	50 (9.9%)	55 (10%)	-	-	25 (4.6%)	28 (5.1%)	24.6	-
Hesham,2024	82	101	29.5 ± 6.0	29.7 ± 5.4	39.2 ± 1.1	39.2 ± 1.1	112 ± 118	120 ± 98	11.8% (8/68)	11.8% (10/85)	100%	100%	1.5% (1/68)	2.4% (2/85)	8.8% (6/68)	9.4% (8/85)
Andre,2024	43	49	29 (3.07)	29.37 (3.44)	39.93 (1.30)	40.30 (0.99)	33.00 (23.01)	33.33 (22.91)	scalp elecrode (36%)	scalp electrode (45%)	-	-	4 (9.3%)	6 (12.2%)	4 (9.3%)	4 (8.2%)
Hoeller,2024	105	104	33(4)	33 (5)	39.857 (1.14)	39.857 (1.14)	130 (58)	131 (68)	-	-	88 (84%)	78 (75%)	-	-	-	-

In contrast, outcomes such as perineal tear grade 2, perineal tear grade 3–4, labial tears, episiotomy rate, and Apgar score < 7 at 5 min were all rated as low evidence certainty. For perineal tear grade 2 (RR 0.92, 95% CI 0.78–1.08), among three RCTs (879 participants), and perineal tear grade 3–4 (RR 0.76, 95% CI 0.47–1.23), among four RCTs (1977 participants), the certainty in both was downgraded to low due to serious inconsistency (I^2^ = 60% and 53%, respectively) and serious imprecision due to the wide confidence interval crossing the line of no effect. For labial tears, the effect estimate resulted from analysis of two RCTs (301 participants) after a “leave-one-out test,” this analysis showed a significant decrease with the group using a perineal protection device compared to the control group (RR 0.72, 95% CI 0.54–0.96; *p* = 0.02) with no heterogeneity (I^2^ = 0%). However, the certainty was downgraded to low due to both serious risk of bias and serious imprecision, as the small sample size and the wide confidence interval. Similarly, for episiotomy rate (RR 0.96, 95% CI 0.81–1.13) across five RCTs (2130 participants), and Apgar score < 7 at 5 min (RR 0.99, 95% CI 0.87–1.13) from four RCTs (1042 participants), were also downgraded to low due to both serious risk of bias and serious imprecision, as the wide confidence interval for the effect estimate crossed the null value, despite the absence of heterogeneity (I^2^ = 0%). The risk of bias for labial tears and episiotomy rate was mainly related to outcome assessment methods between groups, which may have introduced detection bias. For the Apgar score < 7 at 5 min, the risk of bias is mainly due to the outcome assessor being aware of the intervention.

Overall, the certainty of the evidence ranged from moderate (for both outcomes intact perineum and perineal tear grade 1) to low (for all other outcomes), mainly due to inconsistency, imprecision, and risk of bias (Supplementary Table 2).

### Perinium

#### Intact Perineum

**Fig. 3:**
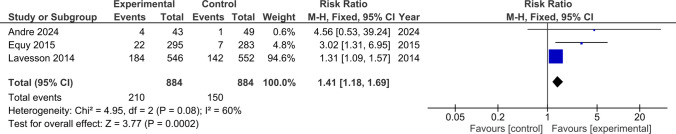
Forest plot illustrating the pooled risk ratio for intact perineum rates. Squares show each study’s risk ratio estimate with 95% confidence intervals, and the diamond represents the overall pooled risk ratio

Three studies involving 1768 participants were included in this analysis. The experimental group showed a statistically significant increase in the rate of intact perineum compared to the control group (RR 1.41; 95% CI 1.18–1.69; *p* < 0.001). Moderate, but insignificant heterogeneity was detected (*p* = 0.08, I^2^ = 60%) (Fig. 3).

#### Perineal Tear

##### Perineal Tear Grade 1

**Fig. 4:**
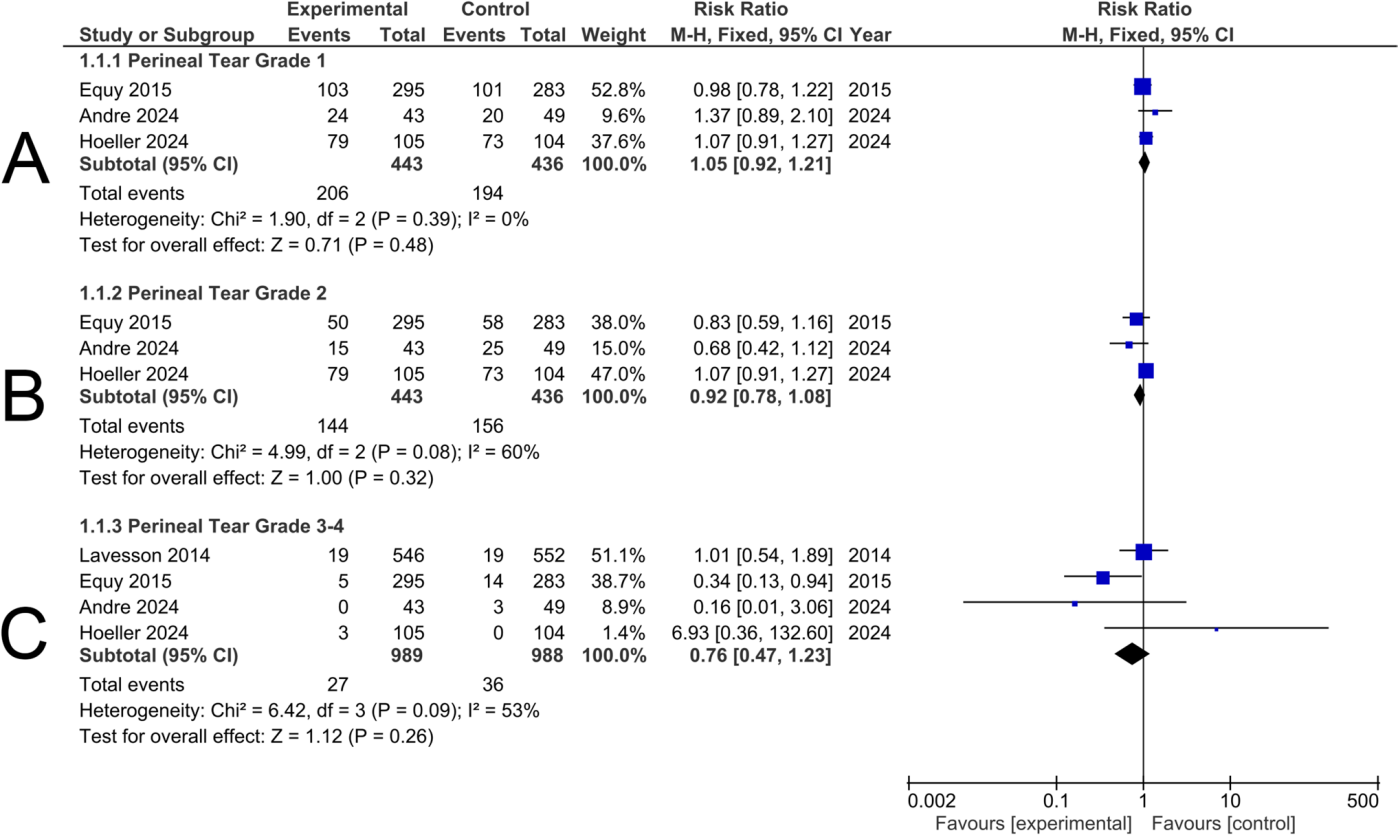
Forest plot showing pooled risk ratios for perineal tear grades: **A** Grade 1, **B** Grade 2, and **(C)** Grades 3–4. Squares show each study’s risk ratio estimate with 95% confidence intervals, and diamonds represent the overall pooled risk ratio for each group

Three studies involving 879 participants were included in this analysis. There was no statistically significant difference regarding grade 1 perineal tears between the experimental and the control groups (RR 1.05; 95% CI 0.92–1.21; *p* = 0.48), and no heterogeneity was detected (*p* = 0.39, I^*2*^ = 0%) (Fig. 4A).

##### Perineal Tear Grade 2

Three studies involving 879 participants were included in this analysis. There was no statistically significant difference regarding grade 2 perineal tears between the experimental and the control groups (RR 0.92; 95% CI 0.78–1.08; *p* = 0.32). Moderate, but insignificant heterogeneity was detected (*p* = 0.08, I^2^ = 60%) (Fig. 4B).

##### Perineal Tear Grade 3–4

Four studies involving 1977 participants were included in this analysis. There was no statistically significant difference regarding grade 3–4 perineal tears between the experimental and the control groups, although it was higher among the control groups (RR 0.76; 95% CI 0.47–1.23; *p* = 0.26). Moderate, but insignificant heterogeneity was detected (*p* = 0.09, I^2^ = 53%) (Fig. 4C).

### Labial Tear

**Fig. 5:**
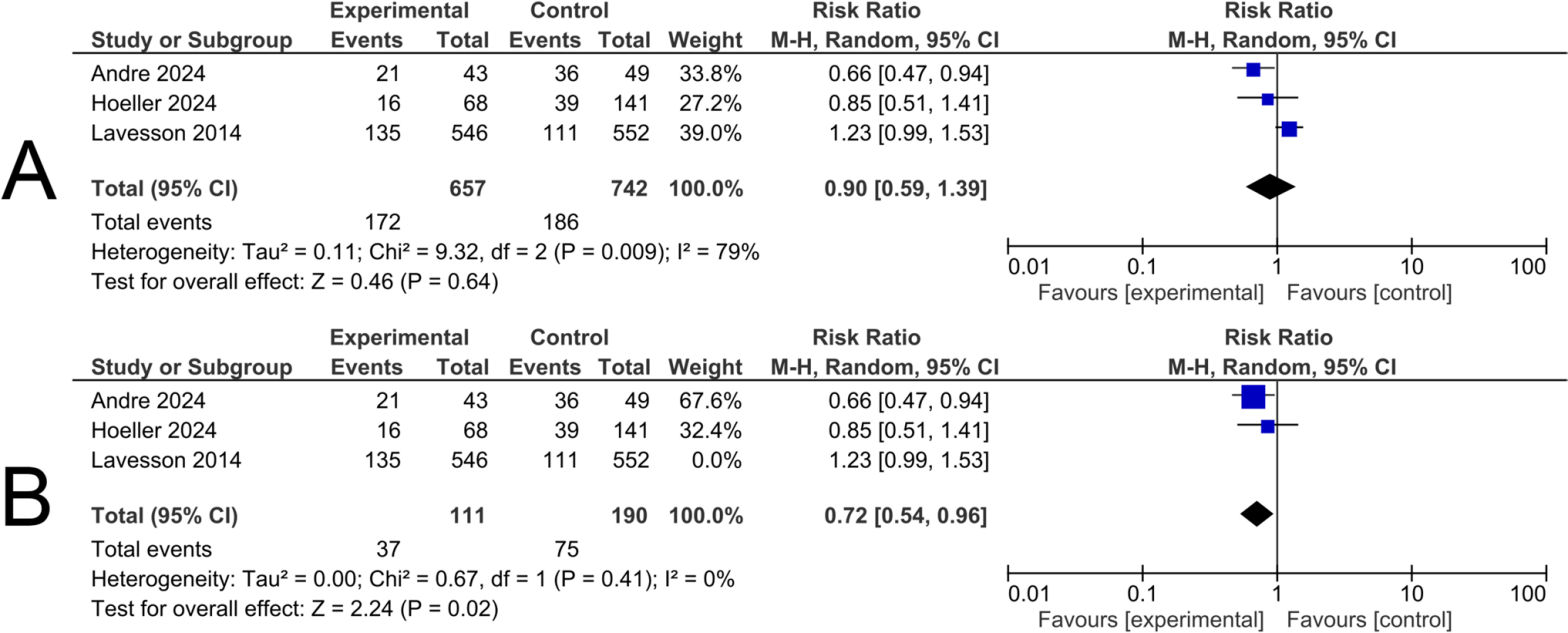
**A** Forest plot illustrating the pooled risk ratio for labial tear rates. Squares show each study’s risk ratio estimate with 95% confidence intervals, and the diamond represents the overall pooled risk ratio. **B** Leave-one-out analysis excluding the study by Lavesson (2014)

Three studies involving 1399 participants were included in this analysis. There was no statistically significant difference regarding the labial tears rate between the experimental and the control groups (RR 0.90; 95% CI 0.59–1.39; *p* = 0.64). Significant heterogeneity was detected (*p* < 0.001, I^2^ = 79%) (Fig. 5A).

A leave-one-out test was conducted to solve the heterogeneity by the study Lavesson 2014, the results showed significant increases in the labial tears rate among the control groups compared to the experimental groups (RR 0.72; 95% CI 0.54–0.96; *p* = 0.02). Heterogeneity was significantly decreased (*p* = 0.41, I^2^ = 0%) (Fig. 5B).

### Episiotomy Rate

**Fig. 6:**
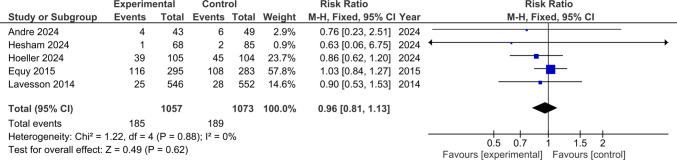
Forest plot illustrating the pooled risk ratio for episiotomy rates. Squares show each study’s risk ratio estimate with 95% confidence intervals, and the diamond represents the overall pooled risk ratio

Five studies involving 2130 participants were included in this analysis. There was no statistically significant difference regarding episiotomy rate between the experimental and the control groups (RR 0.96; 95% CI 0.81–1.13; *p* = 0.62). No significant heterogeneity was detected (*p* = 0.88, I^2^ = 0%) (Fig. 6).

### Apgar Scores (< 7 at 5 min)

**Fig. 7:**
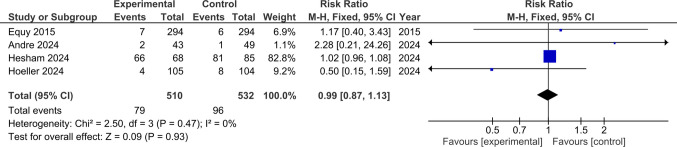
Forest plot showing the pooled risk ratio for Apgar scores < 7 at 5 min. Squares show each study’s risk ratio estimate with 95% confidence intervals, and the diamond represents the overall pooled risk ratio

Four studies involving 1042 participants that reported Apgar scores at 5 min were included in the analysis. No statistically significant difference regarding Apgar scores < 7 at 5 min between experimental and control groups was observed (RR 0.99; 95% CI 0.87–1.13; *p* = 0.93). No significant heterogeneity was detected. (*p* = 0.47, I^2^ = 0%) (Fig. 7).

## Discussion

While to the best of our knowledge, this is the first systematic review evaluating perineal protection devices (PPDs), our study included 2331 women from various geographic regions. The use of perineal protection devices was associated with higher rate of intact perineum compared to manual maneuver. This evidence showed moderate but insignificant heterogeneity. On the other hand, the comparison between PPDs and manual techniques demonstrated no significant effect in perineal tears. However, the evidence was moderately heterogenous in grades 2–4 but was insignificant, whereas no heterogeneity was detected in grade 1. This suggests that PPDs are advantageous in preserving perineal integrity, but they do not necessarily reduce the incidence of perineal tears once they occur. Furthermore, the effect of PPDs showed no significant difference in eliminating labial tears rate with a significant heterogeneity which was resolved by conducting the leave-one-out test. All the included studies evaluated the episiotomy rate which revealed that PPDs have no effect on its rate. Assessing the Apgar scores showed no differences between PPDs and manual equipment, excluding potential adverse neonatal outcomes associated with PPD use.

Regarding the adverse outcomes that can occur during the second stage of labor which affect in the short-term and long-term the maternal complaints, numerous studies were conducted to assess the efficacy of manual techniques or protection devices. In one Cochrane review, results showed no evidence of the manual perineal techniques in reducing perineal traumas or post-partum hemorrhage [28]. In another study, the results suggest evidence of applying some perineal techniques to eliminate third- and fourth-degree perineal tears such as warm compresses and massage without any proven advantages on the other outcomes [29]. Another systematic review has been conducted to evaluate the effects of perineal massage during the antenatal period and second stage of labor on pelvic floor functions and reducing perineal tears in primiparous women. This review showed a positive effect of perineal massage during the second stage of labor in decreasing the rate of episiotomies and second-degree of perineal lacerations, with an elimination in postpartum pain in the early stage. Nonetheless, perineal massage during the antenatal period contributed to reducing postpartum fecal and flatus incontinence at 3 months postpartum [30]. This technique may be considered as an adjuvant procedure to the PPDs to enhance the positive role of PPDs to achieve better outcomes of perineal protection.

Comparing between manual techniques, hands-on and hands-off techniques in spontaneous vaginal deliveries showed no significant effect in reducing severe perineal tears or intact perineum rates. In contrast, the hands-on technique was revealed to increase the third-degree perineal tears, which suggests that using PPDs is associated with better outcomes than the hands-on technique by increasing the intact perineum incidence without significantly affecting the severe perineal traumas rates [31]. Different results among the included studies may be interpreted by the heterogeneity of the included samples as two studies included only a primiparous sample which is accompanied by a higher rate of perineal tears [4, 26, 32]. Another reason is that two of the included studies evaluated the devices during deliveries assisted by vacuums which reported a higher incidence of perineal tears compared to spontaneous births. In addition, the ability of applying PPDs along with the vacuum may disturb the mechanism and lead to worse results and inappropriate application of the device [25, 27].

According to our results, there was moderate-quality evidence suggesting that the use of perineal protection devices increases the rate of intact perineum. Thus, PPH should be considered as a first-line intervention for women with low to moderate risk of perineal trauma to achieve less postpartum pain and improve the recovery process. However, the effectiveness of using these devices has no superior advantage compared to manual techniques in reducing perineal tears, labial tears, and episiotomy rates. Although the effect of PPD was insignificant, the devices reduce grade 3 and grade 4 perineal tears. This result emphasizes the suggestion of applying PPDs particularly in high-risk patients which may reduce the severe tears combined with these cases (e.g., nulliparous, assisted-vaginal delivery, or prolonged second-stage labor). PPDs seem to be safe without harmful neonatal outcomes as the results showed no difference in Apgar scores. We suggest using perineal protection devices during the second stage of labor among high-risk pregnant women for lacerations and tears. However, owing to insufficient data and limited benefits across all outcomes, routine use of PPDs is not currently recommended.

## Limitations and Future Direction

Nevertheless, some limitations require consideration and direct future studies. The variability noted among studies, especially regarding the specific application methods of PPDs, indicates a necessity for enhanced standardization in clinical practice and research methodology. Subsequent research should seek to formulate definitive, generally applicable methods for PPD utilization to reduce outcome variability and enhance the evidence basis. Moreover, although our study included a varied sample, the limited amount of research focusing on multiparous women underscores the gap in the literature. Broadening research to include a wider range of parity groups will improve the generalizability of results and yield a more thorough comprehension of PPD effectiveness among various maternal populations. Another area for future research is improving the evaluation of PPD effectiveness in certain delivery situations. The impact of instrument-assisted births on perineal outcomes necessitates additional clarification. Future studies may benefit from investigations aimed at distinguishing the effects of PPDs in spontaneous vaginal deliveries compared to instrument-aided deliveries, or to examine optimal PPD administration tactics during assisted births. This would elucidate the actual effects of PPDs and mitigate possible confounding variables. Additionally, while our evaluation focused on short-term outcomes, the long-term effects of PPD usage on maternal perineal health and satisfaction remain a relevant subject for future research. Longitudinal trials with prolonged follow-up durations are essential to comprehensively evaluate the enduring benefits and any possible long-term consequences of PPDs, incorporating patient-reported outcomes such as comfort, pain, and sexual function. Lastly, it is important to create and test structured training programs for healthcare workers on how to use PPDs correctly to make sure they are used consistently and effectively in clinical settings. By focusing on these regions, we may not only increase the evidence for PPDs but also make perineal care better and improve maternal outcomes.

## Conclusion

PPDs are effective in increasing the rate of intact perineum, indicating a clear benefit in preventing perineal trauma. While they do not significantly reduce all grades of perineal tears, they appear safe for both mother and neonate and may offer advantages in preventing severe tears in high-risk pregnancies. Additional extensive, standardized trials are necessary to validate the role of PPDs in regular obstetric care, especially in high-risk cases, and to formulate more definitive protocols for their application in clinical practice.

## Supplementary Information

Below is the link to the electronic supplementary material.

Supplementary file1 (DOCX 15 KB)

Supplementary file2 (DOCX 19 KB)

## Data Availability

No data associated with the manuscript.

## Abbreviations

*PPD *: Perineal protection device
*OASIS*: Obstetric anal sphincter injury
*Apgar*: Appearance, pulse, grimace, activity, and respiration (score)
*EAS*: External anal sphincter
*IAS*: Internal anal sphincter
*PPh*: Postpartum hemorrhage
*RR*: Risk ratio
*MD*: Mean difference
*CI*: Confidence interval
*GRADE*: Grading of Recommendations Assessment, Development and Evaluation
*PRISMA*: Preferred Reporting Items for Systematic Reviews and Meta-Analyses
