# Supplementary material for: Effectiveness of Perineal Protection Devices in Reducing Birth-Related Perineal Trauma: A Systematic Review and Meta-Analysis of Randomized Controlled Trials and GRADE Assessment
Source: Int Urogynecol J. 2025 Nov 19;37(2):261–75. doi: 10.1007/s00192-025-06364-y (PMC12995966; doi:10.1007/s00192-025-06364-y)
Supplement: Supplementary file 1 — Supplementary file1 (DOCX 15 KB) [file 192_2025_6364_MOESM1_ESM.docx]

# **Supplementary table 1:** Search strategies for all databases:

| **Database** | **Search strategy** | **Number of citations** |
| --- | --- | --- |
| **PubMed** | ("Perineum"[Mesh] OR perineal OR perineum) AND ("Wounds and Injuries"[Mesh] OR trauma OR tear* OR laceration* OR injury OR "obstetric injury" OR "perineal trauma" OR episiotomy OR "anal sphincter injury") AND ("Protective Devices"[Mesh] OR device* OR "perineal support device*" OR "perineal protection device*" OR "birth support device*" OR "perineal guard" OR "Epi-No" OR "vaginal balloon" OR "silicone ring" OR "perineal distension device") AND ("Labor, Obstetric"[Mesh] OR "Childbirth"[Mesh] OR delivery OR birth OR "vaginal delivery") | 83 |
| **Scopus** | ("Perineum" OR perineal OR perineum) AND ("Wounds and Injuries" OR trauma OR tear* OR laceration* OR injury OR "obstetric injury" OR "perineal trauma" OR episiotomy OR "anal sphincter injury") AND ("Protective Devices" OR device* OR "perineal support device*" OR "perineal protection device*" OR "birth support device*" OR "perineal guard" OR "Epi-No" OR "vaginal balloon" OR "silicone ring" OR "perineal distension device") AND ("Labor, Obstetric" OR "Childbirth" OR delivery OR birth OR "vaginal delivery") | 1353 |
| **Web of Science** | ("Perineum" OR perineal OR perineum) AND ("Wounds and Injuries" OR trauma OR tear* OR laceration* OR injury OR "obstetric injury" OR "perineal trauma" OR episiotomy OR "anal sphincter injury") AND ("Protective Devices" OR device* OR "perineal support device*" OR "perineal protection device*" OR "birth support device*" OR "perineal guard" OR "Epi-No" OR "vaginal balloon" OR "silicone ring" OR "perineal distension device") AND ("Labor, Obstetric" OR "Childbirth" OR delivery OR birth OR "vaginal delivery") | 79 |
| **Embase** | ('Perineum'/exp OR perineal OR perineum) AND ('Wounds and Injuries'/exp OR trauma OR tear* OR laceration* OR injury OR 'obstetric injury' OR 'perineal trauma' OR episiotomy OR 'anal sphincter injury') AND ('Protective Devices'/exp OR device* OR 'perineal support device*' OR 'perineal protection device*' OR 'birth support device*' OR 'perineal guard' OR 'Epi-No' OR 'vaginal balloon' OR 'silicone ring' OR 'perineal distension device') AND ('Labor, Obstetric'/exp OR 'Childbirth'/exp OR delivery OR birth OR 'vaginal delivery') | 308 |
