# Supplementary material for: Effectiveness of Perineal Protection Devices in Reducing Birth-Related Perineal Trauma: A Systematic Review and Meta-Analysis of Randomized Controlled Trials and GRADE Assessment
Source: Int Urogynecol J. 2025 Nov 19;37(2):261–75. doi: 10.1007/s00192-025-06364-y (PMC12995966; doi:10.1007/s00192-025-06364-y)
Supplement: Supplementary file 2 — Supplementary file2 (DOCX 19 KB) [file 192_2025_6364_MOESM2_ESM.docx]

| Outcome | Study design | № of Studies | № of Participants | Risk of Bias | Inconsistency | Indirectness | Imprecision | Publication Bias | Quality of evidence | Effect Estimate | I² | Importance |
| --- | --- | --- | --- | --- | --- | --- | --- | --- | --- | --- | --- | --- |
| Intact perineum | RCTs | 3 | 1768 | no serious | serious | no serious | no serious | no serious | ⊕⊕⊕◯ | RR 1.41 (1.18-1.69) | 60% | important |
| Perineal tear grade 1 | RCTs | 3 | 879 | no serious | no serious | no serious | serious | no serious | ⊕⊕⊕◯ | RR 1.05 (0.92-1.21) | 0% | important |
| Perineal tear grade 2 | RCTs | 3 | 879 | no serious | serious | no serious | serious | no serious | ⊕⊕◯◯ | RR 0.92 (0.78-1.08) | 60% | important |
| Perineal tear grade 3-4 | RCTs | 4 | 1977 | no serious | serious | no serious | serious | no serious | ⊕⊕◯◯ | RR 0.76 (0.47-1.23) | 53% | critical |
| Labial tears | RCTs | 2 | 301 | serious | no serious | no serious | serious | no serious | ⊕⊕◯◯ | RR 0.72 (0.54-0.96) | 0% | important |
| Episiotomy rate | RCTs | 5 | 2130 | serious | no serious | no serious | serious | no serious | ⊕⊕◯◯ | RR 0.96 (0.81-1.13) | 0% | important |
| Apgar scores (<7 at 5 minutes) | RCTs | 4 | 1042 | serious | no serious | no serious | serious | no serious | ⊕⊕◯◯ | RR 0.99 (0.87-1.13) | 0% | critical |
| Certainty of the evidence (GRADE): ⨁⨁⨁⨁ = High certainty of evidence ⨁⨁⨁◯ = Moderate certainty of evidence ⨁⨁◯◯ = Low certainty of evidence ⨁◯◯◯ = Very low certainty of evidence | | | | | | | | | | | | |

Summary of the GRADE certainty of evidence for all outcomes
